# Fibroblast-to-myofibroblast switch is mediated by NAD(P)H oxidase generated reactive oxygen species

**DOI:** 10.1042/BSR20130091

**Published:** 2014-01-14

**Authors:** Lirija Alili, Maren Sack, Katharina Puschmann, Peter Brenneisen

**Affiliations:** *Institute of Biochemistry and Molecular Biology I, Medical Faculty, Heinrich-Heine-University, Düsseldorf, Germany

**Keywords:** MAPK, myofibroblast, NAD(P)H oxidase, reactive oxygen species, TGFβ1, tumour–stroma interaction, AP-1, activating protein-1, CCD, charge-coupled device, CM, conditioned media, DCF, 2′,7′-dichlorofluorescein, DMEM, Dulbecco’s modified Eagle’s medium, DNP, 2,4-dinitrophenyl, FN, fibronectin, GPx, glutathione peroxidase, H_2_O_2_, hydrogen peroxide, H_2_DCF-DA, 2′,7′-dichlorodihydrofluorescein diacetate, HDF, human dermal fibroblasts, HPRT1, hypoxanthine guanine phosphoribosyltransferase, HRP, horseradish peroxidase, MAPK, mitogen-activated protein kinase, MF, myofibroblast, NAD(P)H oxidase, NADH/NADPH oxidase, NGS, normal goat serum, O_2_^.−^, superoxide, ROS, reactive oxygen species, rTGFβ1, recombinant transforming growth factor-beta1, SCL-1, squamous cell carcinoma line, αSMA, alpha smooth muscle actin

## Abstract

Tumour–stroma interaction is a prerequisite for tumour progression in skin cancer. Hereby, a critical step in stromal function is the transition of tumour-associated fibroblasts to MFs (myofibroblasts) by growth factors, for example TGFβ (transforming growth factor beta(). In this study, the question was addressed of whether fibroblast-associated NAD(P)H oxidase (NADH/NADPH oxidase), known to be activated by TGFβ1, is involved in the fibroblast-to-MF switch. The up-regulation of αSMA (alpha smooth muscle actin), a biomarker for MFs, is mediated by a TGFβ1-dependent increase in the intracellular level of ROS (reactive oxygen species). This report demonstrates two novel aspects of the TGFβ1 signalling cascade, namely the generation of ROS due to a biphasic NAD(P)H oxidase activity and a ROS-dependent downstream activation of p38 leading to a transition of dermal fibroblasts to MFs that can be inhibited by the selective NAD(P)H oxidase inhibitor apocynin. These data suggest that inhibition of NAD(P)H oxidase activity prevents the fibroblast-to-MF switch and may be important for chemoprevention in context of a ‘stromal therapy’ which was described earlier.

## INTRODUCTION

Tumour progression is characterized by the local accumulation of extracellular matrix components and connective tissue cells surrounding the tumour cluster, a phenomenon called tumour–stroma interaction [1,2]. Disturbance in stroma, composed of inflammatory cells, small vessels, fibroblastic and myofibroblastic cells, constitutes the desmoplastic reaction, suggested to be essential in the development of the invasion process [3].

The composition of reactive stroma, providing structural and vascular support for tumour growth, resembles that of granulation tissue, and MFs (myofibroblasts) play a critical role in driving both the stromal reaction of physiological wound repair [4,5] and of invasive tumours [6]. The MF has acquired the capacity to express the biomarkers αSMA (alpha smooth muscle actin) and the FN (fibronectin) splice variant ED-A FN [7,8]. In carcinogenesis, a wide variety of different cytokines and growth factors are expressed in tumour–stroma interaction, which stimulate intracellular signal transduction pathways resulting in angiogenesis and tumour growth as well as migration during tumour invasion. Among the autocrine and paracrine acting growth factors involved in molecular processes of tumour**–**stroma interaction, transforming growth factor-beta1 (TGFβ1), a 25kDa homodimeric protein, plays a pivotal role [2,3,9,10].

A paracrine effect of tumour cell-derived TGFβ1 on the down-regulation of gap junctional intercellular communication between stromal fibroblasts was shown earlier, dependent on the generation of ROS (reactive oxygen species) [11,12]. In line with this, TGFβ1 induced an increase in H_2_O_2_ (hydrogen peroxide) levels in human lung fibroblasts, which was abrogated by an inhibitor of NAD(P)H oxidase (NADH/NADPH oxidase) [13]. Furthermore, a TGFβ1-triggered activation of NAD(P)H oxidase initiated apoptosis of fetal rat hepatocytes [14]. Tumour cell-derived TGFβ1 increased the generation of myofibroblastic cells, which promote the invasion of tumour cells in an *in-vitro* 3D model [15] and which can be prevented by redox-active nanoparticles (stromal therapy) [16].

However, the components of the TGFβ1/ROS-initiated downstream signalling pathways resulting in αSMA expression have not been sufficiently identified. Here, we demonstrate two novel findings in TGFβ1-initiated αSMA expression. First of all, TGFβ1 initiates two activity peaks of the NAD(P)H oxidase. Secondly, the second activity peak accompanied by a significant expression of the regulatory subunit p67*^phox^*, is responsible for the ROS-dependent increase in stress-activated kinase expression/activation (especially p38) that is involved in αSMA induction. Interestingly, the NAD(P)H oxidase inhibitor apocynin almost completely abrogated TGFβ1-mediated αSMA expression, whereas the xanthin oxidase inhibitor allopurinol, for example, has no effect. These data give a novel insight into the ROS-dependent signalling leading to MF generation and open up new possibilities for chemoprevention in context of a stromal therapy.

## MATERIALS AND METHODS

Cell culture media [DMEM (Dulbecco's modified Eagle's medium)] were purchased from Invitrogen GmbH and the defined fetal calf serum (FCS gold) was from PAA Laboratories (Linz, Austria). All chemicals including protease as well as phosphatase inhibitor cocktail 1 and 2 were obtained from Sigma or Merck Biosciences unless otherwise stated. The protein assay kit (Bio-Rad DC, detergent compatible) was from Bio-Rad Laboratories GmbH (München, Germany). Apocynin was delivered by Calbiochem. The enhanced chemoluminescence system (SuperSignal West Pico Maximum Sensitivity Substrate) was supplied by Pierce. The Oxyblot Protein Oxidation Detection kit was from Millipore. The dye H_2_DCF-DA (2′,7′-dichlorodihydrofluorescein diacetate) was supplied from MoBiTec. PCR primers were synthesized by Invitrogen. Reagents for SDS–PAGE were from Roth. Monoclonal mouse antibody raised against human αSMA and αTubulin was supplied by Sigma. Polyclonal rabbit antibody raised against human phospho P38 was supplied by New England Biolabs. The following secondary antibodies were used: polyclonal HRP (horseradish peroxidase)–conjugated rabbit anti-mouse IgG antibody (DAKO), goat anti-rabbit immunoglobulin G antibodies were from Dianova and Alexa Fluor 488-coupled goat anti-mouse IgG antibody (H+L) (MoBiTec GmbH). rTGFβ1 (recombinant human TGFβ1) was delivered by R&D Systems.

### Cell culture

HDF (human dermal fibroblasts) were established by outgrowth from foreskin biopsies of healthy human donors aged from 3–6 years. Cells were used in passages 2–11, corresponding to cumulative population doubling levels of 3–23 [17]. Dermal fibroblasts and the SCL-1 (squamous cell carcinoma line), originally derived from the face of a 74-year-old woman [18] (generously provided by Professor Dr Norbert Fusenig, DKFZ Heidelberg, Germany) were maintained in DMEM supplemented with glutamine (2 mM), penicillin (400 units/ml), streptomycin (50 μg/ml) and 10% (v/v) FCS in a humidified atmosphere of 5% (v/v) CO_2_ and 95% (v/v) air at 37°C. MFs were generated by treatment of HDFs with rTGFβ1 for 48 h in HDF conditioned medium (CM^HDF^) [15].

### Preparation of conditioned media (CM)

CM was obtained from SCL-1 cells (CM^SCL^) and HDF (CM^HDF^). SCL-1 cells at an initial density of 1×10^6^ cells were grown to subconfluence (~70% confluence) and 1,5×10^6^ HDF cells to confluence in 175 cm^2^ culture flasks to get identical cell numbers. The serum containing medium was removed, and after washing three times in PBS the cells were incubated for further 48 h in 15 ml serum-free DMEM before collection of the CM.

### RNA isolation and quantitative real-time RT–PCR

Total RNA was isolated and transcribed into cDNA as described. mRNA levels were analysed by RT–PCR either by using a Thermocycler (Biometra) as described in [19] or in the LightCycler system (Roche). Real-time RT–PCR was performed with 40 ng cDNA in glasscapillaries containing LightCycler FastStart DNA Master SYBR Green I Reaction Mix (Roche), 2 mM MgCl_2_ and 1 μM of primers. Quantification of the PCR amplicons was performed using the LightCycler Software. HPRT1 (hypoxanthine guanine phosphoribosyltransferase) was used as internal normalization control [20]. Sequences of primer pairs are given in Table 1.

**Table 1:** Sequences of primers for RT–PCR

Genes	Primer (5′→3′)
HPRT1	Forward: ATTCTTTGCTGACCTGCTGGATT
	Reverse: CTTAGGCTTTGTATTTTGCTTTTC
αSMA	Forward: CTGTTCCAGCCATCCTTCAT
	Reverse: TCATGATGCTGCTGTTGTAGGTGGT
p67*^phox^*	Forward: CGAGGGAACCAGCTGATAGA
	Reverse: CATGGGAACACTGAGCTTCA
NOX4	Forward: GAAGCCCATTTGAGGAGTCA
	Reverse: GGGTCCACAGCAGAAAACTC

### Measurement of intracellular ROS

The intracellular ROS level was measured using the fluorescent dye H_2_DCF-DA, which is diffusible into cells and there hydrolysed to the non-fluorescent derivative H_2_DCF [12]. In the presence of peroxides, H_2_DCF is converted into the highly fluorescent DCF (2′,7′-dichlorofluorescein). For assays, subconfluent fibroblast monolayer cultures were loaded with 20 μM H_2_DCF-DA in PSG buffer (100 mM KH_2_PO4, 10 mM NaCl, and 5 mM glucose; pH 7.4) for 15 min in the dark. After washing three times with PSG buffer, the loaded cells were subjected to 10 ng rTGFβ1/ml PSG. ROS generation was detected as a result of the oxidation of H_2_DCF and the fluorescence (excitation 488 nm; emission 515–540 nm), given in arbitrary units, was followed with a Zeiss axiovert fluorescent microscope with a CCD (charge-coupled device) camera (ORCA II, Hamamatsu) for 20 min.

### Determination of extracellular H_2_O_2_

An extracellular concentration of H_2_O_2_was quantified by amperometric determination using the Apollo4000 (Worldprecision Instruments) with the H_2_O_2_sensor ISO-HPO-2 sensor tip according to the manufacturer's instruction. Briefly, a calibration curve was generated by the injection of different amounts of H_2_O_2_to the calibration solution (PBS). Plotting of the changes in current (pA) against the changes in concentration (nM) creates a calibration curve. For measuring extracellular H_2_O_2_concentration, 50 μl of cell supernatants were injected to the calibration solution and current changes were recorded. Although, the sensitivity of the sensor does not change significantly within in the temperature range of 20–37°C, all measurements and generation of the calibration curve were done at 37°C.

### Measurement of NAD(P)H oxidase activity

NAD(P)H oxidase has the E.C. number 1.6.1.3 reflecting one enzyme that uses both NADH and/or NADPH oxidase as substrate and O_2_ as electron acceptor. Therefore the term NAD(P)H oxidase is often used–and we like to do it as well–to represent both possible substrates. However, as the *K*_m_ value for NADPH is lower than for NADH, the endogenous NAD(P)H oxidase would prefer NADPH.

Nevertheless, many publications used NADH as substrate for the cell-free activity measurements as we did as well (see Figure 4A). Here, we like to discriminate between ‘NADH oxidase activity’ and ‘NAD(P)H oxidase’ (meaning the enzyme in general).

In this paper, the NADH oxidase activity of the NAD(P)H oxidase was measured. Fibroblasts were grown to 70% confluence and washed with prewarmed HBSS (Hank's buffered salt solution). After 15 min incubation with 10 ng rTGFβ1/ml or mock treatment in serum-free medium, cells were exposed to 250 μM NADH/HBSS or NADPH/HBSS for 1 min. The rate of NADH/NADPH consumption was measured as decrease in absorbance at 340 nm using a spectrophotometer (Ultrospec 1000, Pharmacia Biotech). The extinction coefficient for calculation of the concentration of consumed NADH/NAD(P)H was 6.22 mM^−1^ cm^−1^. For measurements of the specific NAD(P)H oxidase activity, herein the rate of NADH consumption inhibitable by apocynin, a specific NAD(P)H oxidase inhibitor was used as described earlier [13]. Data were expressed in nmol NADH consumption min^−1^ mg^−1^ protein.

### Immunocytochemistry

HDF monolayer cultures were grown in DMEM plus 10% (v/v) FCS on coverslips in 3.5 cm diameter tissue culture dishes before use. Cells were washed with PBS and fixed with methanol for 10 min at 4°C. After washing with PBS, non-specific binding of antibodies was blocked with 3% (v/v) NGS (normal goat serum) in TBST containing 0.3% (v/v) Triton X-100 at room temperature (20°C). Cells were incubated with monoclonal αSMA antibody diluted 1:1000 in 1% (v/v) NGS/TBST overnight at 4°C. After washing the cells were incubated with an Alexa 488-coupled goat anti-mouse IgG (1/1000 diluted in TBST) for 1 h at room temperature. For DAPI staining, cells were incubated for 10 min at room temperature with 1:500 diluted DAPI solution (Sigma, stock solution 0.5 mg/10 ml H_2_O) in McIlvaine's buffer (100 mM citric acid, 200 mM Na_2_HPO_4_; pH 7.2). After washing and embedding, images were taken with a Zeiss Axiovert fluorescence microscope with a CCD camera.

### SDS–PAGE and Western blotting

SDS–PAGE was performed according to the standard protocols published elsewhere [21], with minor modifications. Briefly, cells were lysed after incubation with rTGFβ1 (10 ng/ml) in 1% (w/v) SDS with 1:1000 protease inhibitor cocktail (Sigma). After sonication, the protein concentration was determined by using a modified Lowry method (Bio-Rad DC). 4x SDS–PAGE sample buffer [1.5 M Tris–HCl (pH 6.8), 6 ml 20% SDS, 30 ml glycerol, 15 ml β-mercaptoethanol and 1.8 mg bromophenol blue] was added, and after heating, the samples (10–30 μg total protein/lane) were applied to 8–15% (w/v) SDS–PAGE. After electroblotting onto PVDF membrane (GE Healthcare), immunodetection was carried out using an 1:1000 dilution of primary antibodies (mouse monoclonal anti αSMA and α-tubulin or rabbit monoclonal anti phospho p38), 1:20000 dilution of anti-mouse/rabbit antibody conjugated to HRP). Antigen–antibody complexes were visualized by an enhanced chemiluminescence system. α-tubulin or Coomassie Brilliant Blue staining was used as internal control for equal loading. Molecular sizes of the bands were calculated by comparison with a prestained protein marker (Fermentas, St. Leon-Rot). For quantification of the bands, the developed films were scanned by an image analysis system and analysed with the ImageJ software.

### Determination of oxidized (carbonylated) proteins

HDF were grown to subconfluence on tissue culture dishes. After removal of serum-containing medium, cells were cultured in the serum-free medium and either mock-treated or treated for 24 h with TGFβ1 (10 ng/ml). As a positive control, the cells were treated with H_2_O_2_ (1 mM) for 1 h. Thereafter, cells were lysed and carbonyl groups of oxidized proteins were detected with the OxyBlot™ Protein Oxidation Detection Kit, following the manufacturer's protocol. Briefly, the protein concentration was determined by using a modified Lowry method (Bio-Rad DC). Roughly, 5 μg of the cell lysates were incubated with DNP (2,4-dinitrophenyl) hydrazine to form the DNP hydrazone derivatives. Labelled proteins were separated by SDS–PAGE and immunostained using rabbit anti-DNP antiserum (1:500) and goat anti-rabbit IgG conjugated to HRP (1:2000). Blots were developed by enhanced chemiluminescence.

### Statistical analysis

Means were calculated from at least three independent experiments, and error bars represent standard error of the mean (S.E.M.). Analysis of statistical significance was performed by Student's *t* test or ANOVA with *P<0.05, **P<0.01 and ***P<0.001 as the levels of significance.

## RESULTS

### Recombinant- and tumour cell-derived TGFβ1 induce fibroblast-to-MF transition

**Figure 1:**
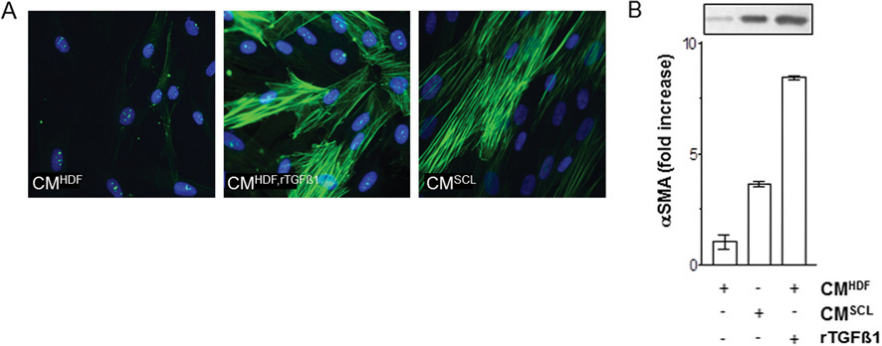
TGFβ1-mediated transition of fibroblasts to MFs Subconfluent HDF were either mock-treated (CM^HDF^), treated with rTGFβ1 (10 ng/ml) for 48 h (CM^HDF,TGF^) and in CM of squamous carcinoma cells SCL-1 (CM^SCL−1^). (**A**) The amount of αSMA protein was immunostained for αSMA and (**B**) determined by Western blot analysis. The densitometric values represent the fold increase over control, which was set at 1.0. The data represent means±S.E.M. of three independent experiments. CM, conditioned medium.

Immunocytochemistry studies show a significant increase in αSMA staining after treatment with rTGFβ1 (CM^HDF, rTGFβ1^) compared with mock-treated cells (CM^HDF^). Furthermore, a significant amount of active TGFβ1-containing CM of SCL-1 tumour cells (CM^SCL^) [12] resulted in formation of MFs as well (Figure 1A). The staining reveals the organization of αSMA in stress fibres, a morphological property of MFs.

In order to evaluate an *x*-fold increase in TGFβ1-triggered expression of αSMA (CM^HDF, rTGFβ1^; CM^SCL^) in comparison with mock-treatment (CM^HDF^), subconfluent HDF were treated with recombinant- and tumour cell-derived TGFβ1. Treatment with both rTGFβ1 and CM^SCL^ resulted in an about 9-fold and 4-fold increase of αSMA expression, respectively (Figure 1B). As CM^SCL^ and rTGFβ1 show the similar results, rTGFβ1 was used for the further experiments.

### Effect of allopurinol, apocynin and DPI on TGFβ1 induced αSMA expression

The growth factor TGFβ1 was shown to be involved in production of ROS, especially O_2_^.−^ (superoxide) and H_2_O_2_ [12,15,22,23].

**Figure 2:**
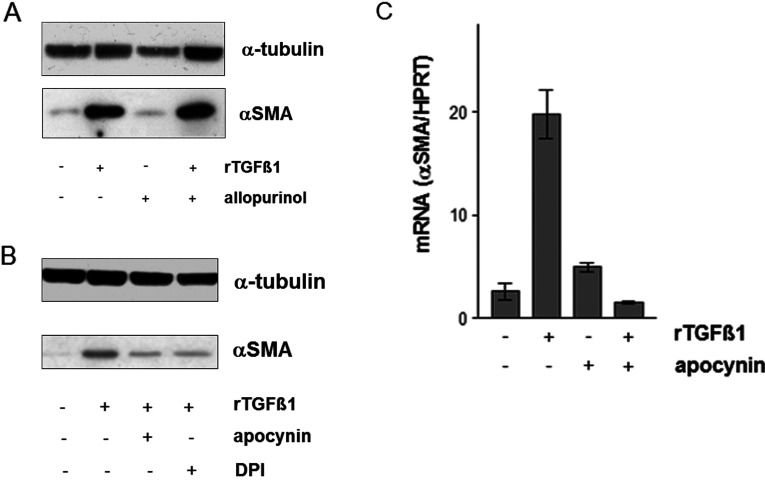
TGFβ1-mediated expression of αSMA (**A**) Subconfluent HDFs were either mock-treated or pretreated for 24 h with allopurinol (10 μM) before addition of rTGFβ1 (10 ng/ml). TGFβ1 and the allopurinol were present for an additional 48 h. The level of αSMA protein was determined by Western blot. α-tubulin was used as loading control. Three independent experiments were performed. (**B**) HDF monolayer cultures were cultured in CM^HDF^ containing apocynin (1 mM) for 1 h or DPI (5 μM) for 24 h before treatment with TGFβ1 (10 ng/ml) for further 48 h. The level of αSMA protein was determined by Western blot. α-tubulin was used as loading control. The experiments were performed in triplicate. (**C**) Subconfluent HDF were preincubated for 1 h with apocynin (1 mM) in serum-free medium and then TGFβ1 (10 ng/ml) treated for 24 h. Steady-state mRNA levels of αSMA were analysed by real-time RT-PCR. Data are given as means of three independent experiments±S.E.M.

Pharmacological approaches using ROS level-modulating substances such as selenite, butylated hydroxytoluene and the vitamin E-derivate Trolox clearly demonstrated a TGFβ1-mediated generation of ROS [12,15], which was prevented by that antioxidants. Selenite and the GPx (glutathione peroxidase) mimic ebselen inhibited the TGFβ1 initiated αSMA expression dealing with GPx to play a major role in that context [15]. An involvement of TGFβ1-initiated higher ROS level mediating down-regulation of gap junctional intercellular communication [12] and expression of αSMA [15] in dermal fibroblasts was demonstrated as well as a TGFβ1-dependent activation of NADH oxidase in lung fibroblast [24]. To address the question of whether NAD(P)H oxidase alone or other major O_2_^.−^/H_2_O_2_ sources such as xanthine oxidase play a role in the transition of fibroblasts to MFs, HDF were exposed to rTGFβ1 in the presence and absence of non-toxic concentration of allopurinol (10 μM), apocynin (1 mM) and DPI (5 μM). The effect of the xanthine oxidase inhibitor, allopurinol, on the expression of αSMA was examined. At a dose that has been previously reported to inhibit the xanthine oxidase, allopurinol did not affect the αSMA expression (Figure 2A). Therefore, αSMA expression is independent on xanthine oxidase.

A significant increase in the αSMA protein amount was measured at 48 h on treatment with rTGFβ1 compared with mock-treated controls. This increase was nearly completely abolished by preincubation of the cells with the NAD(P)H oxidase inhibitors apocynin (1 mM) or DPI (5 μM) (Figure 2B). Apocynin, a methoxy-substituted catechol and used as selective inhibitor of NAD(P)H oxidase, inhibits NAD(P)H oxidase by impeding the assembly of p47*^phox^* and p67*^phox^* subunits within the membrane-associated NAD(P)H oxidase complex [25]. Newly, apocynin was shown to have a high capacity as a scavenger of H_2_O_2_ in addition to its function as NOX inhibitor [26]. Apocynin and DPI alone had no effect on αSMA expression compared with mock-treated controls (data not shown). As the inhibition of TGFβ1-mediated αSMA expression by apocynin is significant, further experiments focus on NAD(P)H oxidase and its downstream signalling resulting in MF formation.

To study the effect of apocynin on levels of steady-state mRNA of αSMA in HDF, real-time RT–PCR was performed. The ‘housekeeping’ gene HPRT was used as internal control. TGFβ1 caused a 20±2-fold increase in αSMA steady-state mRNA levels at 24 h after the treatment compared with mock-treated controls. Preincubation with apocynin (1 mM) completely abolished the TGFβ1-mediated increase in the steady-state mRNA level of αSMA (Figure 2C). These data correlated with the αSMA protein amount (Figure 2B).

### Modulation of ROS generation and protein oxidation by apocynin

To test a direct effect of apocynin on ROS production in the fibroblasts, the ROS generation was assessed both intracellularly and extracellularly.

**Figure 3:**
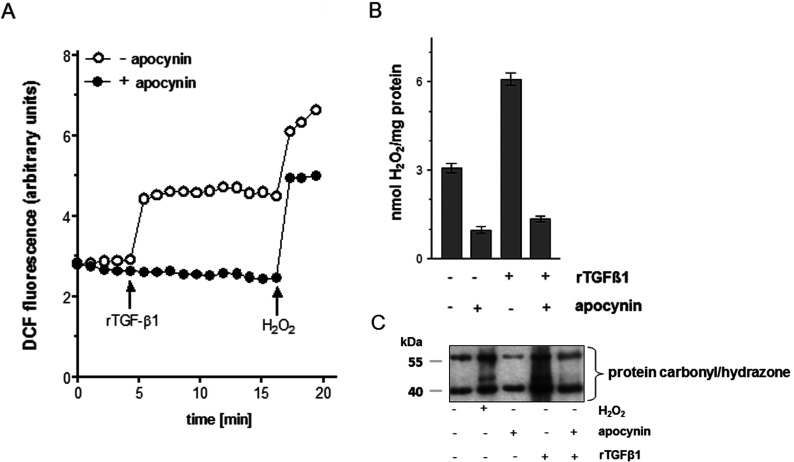
Apocynin inhibits the ROS production and the oxidative damage in HDF (**A**) Subconfluent HDFs were preincubated with apocynin (1 mM) for 1 h (closed circles) before treatment with rTGFβ1 or H_2_O_2_ (1 mM) for the indicated time. Increase of DCF fluorescence was followed over 20 min versus mock-treated controls (open circle). The experiments were performed in triplicate. Arrows indicate addition of rTGFβ1 or H_2_O_2_.(**B**) H_2_O_2_was detected by amperometric determination. The data represent the mean±S.E.M. of three independent experiments. (**C**) HDF cells were exposed to rTGFβ1 for 24 h or preincubated with apocynin (1 mM) before oxidized proteins were detected by Western blot analysis via derivatization with DNP hydrazine. H_2_O_2_ was used as positive control. Three independent experiments were performed.

Incubation with the growth factor TGFβ1 resulted in a significant 2-fold increase in dichlorofluorescein (DCF) fluorescence which was maintained over the studied time range. A non-toxic concentration of 1 mM H_2_O_2_, used as a control, further increased the intracellular ROS level. Preincubation of HDFs with a non-toxic concentration of the specific NAD(P)H oxidase inhibitor apocynin (1 mM) (Figure 3A) prior to TGFβ1 stimulation prevented the growth factor-initiated increase in the ROS level, indicating that generation of elevated ROS levels is downstream of activation of NAD(P)H oxidase and is affected by apocynin. H_2_O_2_ treatment of cells, preincubated with the apocynin and TGFβ1, resulted in a significant increase in DCF fluorescence (Figure 3A).

A potential extracelluar increase in H_2_O_2_should be measured by an amperometric approach, which is highly sensitive for the determination of extracellular H_2_O_2_. TGFβ1 exposure resulted in a significant increase in H_2_O_2_generation (Figure 3B). At 24 h after treatment of HDF cells with TGFβ1, the H_2_O_2_ level was 2-fold higher compared with mock-treated controls. As the production of H_2_O_2_ by TGFβ1 needs a O_2_^.−^ source [27], the effect of apocynin on the production of H_2_O_2_ was measured. Preincubation of fibroblasts for 1 h with apocynin prior to rTGFβ1 treatment down-regulated the TGFβ1-mediated H_2_O_2_generation. However, it is evident that TGFβ1 exposure results in a solid generation of H_2_O_2_. Interestingly, the incubation with apocynin alone inhibited the H_2_O_2_ generation compared with mock-treated control (Figure 3B).

Another, more indirect approach to measure the intracellular generation of ROS, the occurrence of carbonylated proteins, a biomarker for intracellular oxidative stress, was investigated. For that, HDF were treated with TGFβ1 and the carbonylated proteins verified. A low amount of carbonylated proteins was detected in mock-treated cells, whereas the amount was significantly increased in H_2_O_2_–and TGFβ1-treated cells compared with mock-treated cells (Figure 3C). Treatment with apocynin significantly lowered the TGFβ1-mediated protein oxidation. H_2_O_2_was used as positive control. Even though the occurrence of protein carbonyls is proof for oxidative stress, the measurement of those carbonyls is rather a general measure of an alteration of the cellular redox status.

### TGFβ1 stimulates a rapid increase in the NAD(P)H oxidase activity

**Figure 4:**
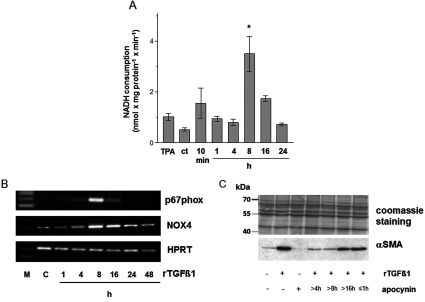
rTGFβ1 activates the NADH oxidase in dermal fibroblasts (**A**) Rates of NADH consumption by ct and time course of the rates from HDF following rTGFβ1 (10 ng/ml) treatment. TPA was used as a positive control. In presence of 250 μM NADH, subconfluent HDF were either mock-treated or treated with rTGFβ1. The consumption of NADH was measured spectrophotometrically. data represent the mean±S.E.M. (**B**) Subconfluent HDF were preincubated for 1 h with apocynin (1 mM) in the serum-free medium and then rTGFβ1 (10 ng/ml) treated for various time points. p67*^phox^* and NOX4 mRNA expression were analysed by RT–PCR. HPRT1 was used as housekeeping gene. Three independent experiments were performed. (**C**) Subconfluent HDFs were either mock-treated, treated with rTGFβ1 (10 ng/ml) for 48 h or incubated with apocynin for 1 h or starting 4, 8 and 16 h after rTGFβ1 treatment. The level of αSMA protein was determined by Western blot. Coomassie Brilliant blue staining was used as loading control. Three independent experiments were performed.

The rates of NADH consumption by control and TGFβ1-stimulated cells were determined at various time points over a 24 h period. TPA (12-O-tetradecanoylphorbol-13-acetate) was used as a positive control. As shown in Figure 4(A), the rate of NADH consumption in TGFβ1-treated cells resulted in two peaks. After 10 min the NADH consumption in TGFβ1-treated cells was 2-fold higher than that of ct (control cells), with no measurable increase at 1 and 4 h. A second peak of NADH consumption was detected at 8 h with a 7-fold increase of NADH consumption compared to ct (*P*<0.05). The treatment with TGFβ1 led to a gradual decrease to baseline (undetectable levels) by 24 h.

In most cell types, the members of the NOX family are the source for the occurrence of ROS, namely superoxide. NAD(P)H oxidase consists of membrane-associated and cytosolic subunits. There are five human NAD(P)H oxidases, namely NOX1 to NOX5 and several cytosolic and regulatory subunits, e.g. p67*^phox^*[28]. It is described, that NOX4 is involved in TGFβ1-mediated differentiation of human cardiac fibroblasts to MFs [29]. In this study, we checked whether rTGFβ1 is involved in expression of genes encoding components of NAD(P)H oxidases. Therefore, the cytosolic subunit p67*^phox^* and NOX4 mRNA was estimated by RT–PCR. cDNA integrity was checked simultaneously by amplification of the housekeeping gene HPRT1. The expression of NOX4 and p67*^phox^* were up-regulated in TGFβ1-treated cells after 8 h (Figure 4B). These data confirm the previously shown NADH consumption peak after 8 h of TGFβ1 treatment (Figure 4A).

Even though the increase of p67*^phox^* and NOX4 mRNA levels (Figure 4B) and the high activity of NAD(P)H oxidase(Figure 4A) both at 8 h deal with the importance of that 8 h peak in context of αSMA expression, it could not be excluded that the first peak at 10 min post-treatment (Figure 4A) affects the αSMA expression as well.

Therefore different incubation periods with apocynin should solve the problem. HDF were exposed to rTGFβ1 in the presence (+) and absence (−) of apocynin (1 mM). A significant increase in the αSMA protein amount was measured 48 h upon treatment with rTGFβ1 compared with mock-treated controls. Apocynin treatment (+) over the total time period of 48 h after TGFβ1 incubation completely abolished the αSMA signal. The incubation with apocynin starting 4 and 8 h after TGFβ1 treatment showed a marked lowering of αSMA expression as well. However, apocynin treatment starting 16 h after TGFβ1 incubation did not affect the αSMA expression (Figure 4C). Furthermore, apocynin incubation during the first hour after TGFβ1 treatment also showed no inhibitory effect on αSMA expression. Thus, the first NAD(P)H oxidase peak seems to play a rather minor role in αSMA signalling. In summary, the NAD(P)H oxidase is essential for αSMA signalling in a time period of 4–8 h after TGFβ1-stimulation.

### Effects of MAPK (mitogen-activated protein kinases) on MF formation

**Figure 5:**
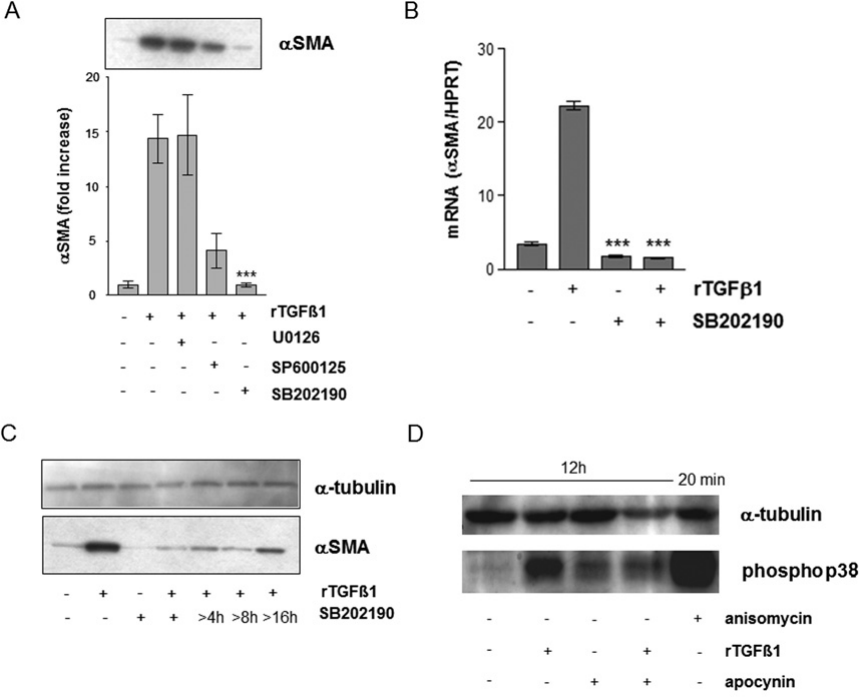
Involvement of p38 kinase in TGFβ1/ROS-dependent expression of αSMA (**A**) Subconfluent HDFs were preincubated with MAPK inhibitors U0126, SP600125 or SB202190 before treatment with rTGFβ1. Expression of αSMA was detected by Western blots. The densitometric analysis describes protein expression as fold increase over control, which was set at 1.0. The data represent the mean±S.E.M. of three independent experiments. (**B**) Subconfluent HDF were preincubated for 1 h with SB202190 (10 μM) in the serum-free medium and then rTGFβ1 (10 ng/ml) treated for 24 h. αSMA mRNA levels were analysed by real-time RT-PCR. Data are given as means of three independent experiments±S.E.M. (**C**) Subconfluent HDFs were either mock-treated, treated with rTGFβ1 (10 ng/ml) for 48 h or incubated with SB 202190 for 48 h or starting 4, 8 and 16 h after rTGFβ1 treatment. The level of αSMA protein was determined by Western blot. α-tubulin was used as loading control. Three independent experiments were performed. (**D**) Subconfluent HDFs were either mock-treated or pretreated for 1 h with apocynin (1 mM) before addition of rTGFβ1 (10 ng/ml). TGFβ1 and apocynin were present for an additional 12 h. Anisomycin (0.5 μg/ml) was used as technical control and incubated for 20 min. The level of phospho-p38 MAPK protein was determined by Western blot. α-tubulin was used as loading control. Two independent experiments were performed.

In fibroblasts of adventitia from vascular cells, ROS generated by NAD(P)H oxidase, activated the MAPK and finally the fibroblasts differentiated to MFs [30]. To check the importance of MAPK during the transition process of HDF, HDF were exposed to rTGFβ1 in the presence and absence of non-toxic concentration of U0126, SP600125 and SB202190 (10 μM). Mock-treated ct showed a basal αSMA expression. A significant increase in the αSMA protein amount was measured at 48 h upon treatment with TGFβ1 compared with mock-treated controls. In our study, JNK (c-Jun N-terminal kinase) inhibitor SP600125 and p38 MAPK inhibitor SB202190 significantly (*P*<0.001) attenuated TGFβ1-induced αSMA expression when added to the cultures 1 h prior to TGFβ1. The inhibitor of ERK (extracellular-signal-regulated kinase) activation U0126 had no effect on αSMA expression (Figure 5A). As the specific p38-inhibitor S 202190 had the most inhibitory effect on αSMA protein level, the effect on αSMA mRNA expression was tested. In the following, the focus was on the p38 kinase and the chronological involvement of p38 in the αSMA signalling. Preincubation with non-toxic concentrations of the p38 MAPK inhibitor significantly (*P*<0.001) counteracted the TGFβ1-initiated transcription of αSMA mRNA (Figure 5B), indicating a crucial role of p38 kinase in the signalling pathway, which results in αSMA expression and MF formation. In the following, a time course analysis after stimulation with rTGFβ1 was performed. The cells were co-incubated with the specific p38 inhibitor SB202190 for different time periods. Mock-treated ct showed a basal αSMA expression. After stimulation with rTGFβ1 the αSMA protein amount increased. The αSMA protein level of cells co-incubated either with rTGFβ1 and SB 202190 over the total time period or with rTGFβ1 and p38 inhibitor starting 4 and 8 h after rTGFβ1 treatment was nearly completely abolished. By contrast, incubation with the inhibitor starting 16 h after rTGFβ1 stimulation showed only a slight but not significant inhibitory effect on αSMA expression, keeping in mind the α-tubulin loading control (Figure 5C). Herein, we have shown that apocynin as well as the p38 inhibitor SB202190 inhibit the TGFβ1-mediated αSMA expression and consequently the MF formation. TGFβ1 generates ROS because of NAD(P)H oxidase, which activates p38 and further stimulates αSMA signalling. The link between the NAD(P)H oxidase and p38 was investigated using apocynin. Incubation with Anisomycin for 20 min showed a distinct signal, the mock-treated fibroblasts showed a weak activation of p38. Treatment with rTGFβ1 for 12 h induced a significant p38 phosphorylation. Preincubation with apocynin (1 mM) nearly completely abolished the increase of the activated p38 (Figure 5D).

## DISCUSSION

**Figure 6:**
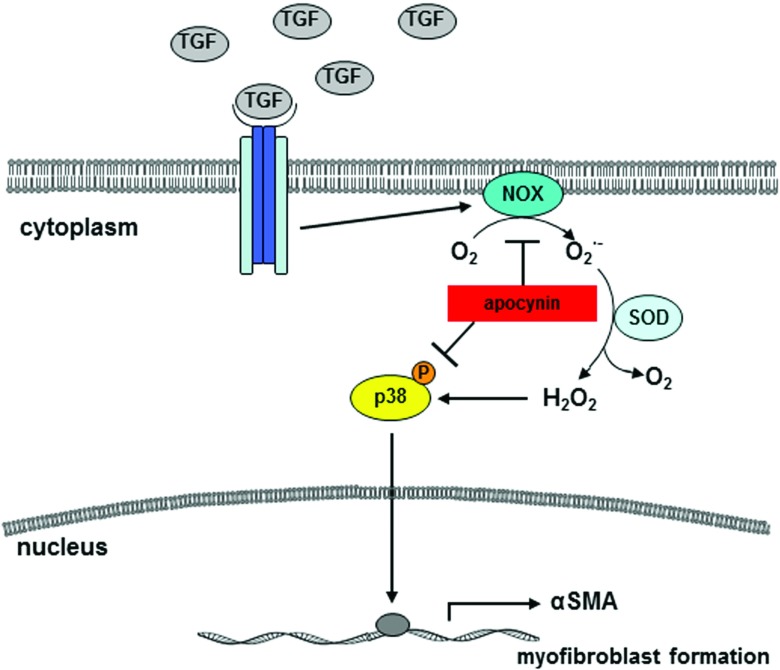
Scheme of TGFβ1-mediated signalling Tumour cells release growth factors, e.g. TGFβ1, which generates ROS due to NAD(P)H oxidase, which is responsible for the downstream signalling resulting in ROS-triggered activation of the stress kinase p38 and expression of αSMA. Both can be inhibited by the specific NAD(P)H oxidase inhibitor apocynin.

The first crucial step in tumour invasion and metastasis is the movement of cancer cells into the tumour-surrounding tissue. Recent data brought prominence to the hypothesis of a role for tumour stromal environment as a leading player, and not just a supporting additional, in the progression of carcinomas, the most common form of human cancer. Fibroblasts have a more profound influence on the development and progression of carcinomas than previously appreciated [1,2]. In that context, MFs and cancer cells are known to exchange proteolytic enzymes, cytokines and growth factors, which promote proliferation and survival as well as invasion of the tumour [31]. In this study, we have shown *in vitro* that the NAD(P)H oxidase is responsible for the downstream signalling resulting in ROS triggered activation of the stress kinase p38 and expression of αSMA after TGFβ1 treatment (Figure 6).

Fibroblasts can be activated by various growth factors and cytokines, which are secreted by tumour cells [15,32,33], and show then myofibroblastic differentiation [34,35]. There is a dynamic interaction between malignant cells and stromal tissue, which is mediated by cell–cell- and cell–matrix communiacations [36]. In our study, the mesenchymal–mesenchymal transition was induced by rTGFβ1 at concentrations ranging from 5 to 10 ng/ml. Recently, similar concentrations of rTGFβ1 were shown to be sufficient to increase αSMA protein levels in rat proximal tubular epithelial cell line NRK52E [37] as well as in human fetal lung fibroblasts [38]. Moreover, these cells showed significantly higher amounts of αSMA protein at 24–48 h after TGFβ1 treatment, which corresponds to our study. Herein, the incubation of HDF with supernatants of squamous tumour cells resulted in a significant increase in αSMA expression as well. The lower effect of the tumour cell supernatants on αSMA expression is due to the lower concentration of active TGFβ1 in the supernatant compared with the activity of rTGFβ1 [12].

The supernatant of SCL-1 tumour cells and TGFβ1 triggered a rapid increase in intracellular ROS levels in HDF leading to an impaired gap junctional intercellular communication [11], a prerequisite for tumour progression [39]. Cellular structures during signalling as well as transcription factors can be modified by TGFβ1 induced ROS [37,40]. As several cellular mechanisms result in the production of ROS [41,42], we asked for the source of ROS. Some studies show the growth factor-/cytokine-dependent generation of ROS during physiological signalling due to membrane-bound enzyme complexes, e.g. the NAD(P)H oxidase that generate O_2_^.−^/H_2_O_2_ [43–45]. TGFβ1 is known to activate NAD(P)H oxidase, resulting in the generation of ROS, which promotes carcinogenesis [46]. As TGFβ1 is known to induce the formation of MFs [3], we addressed the question of whether NAD(P)H oxidase alone or other sources such as xanthine oxidase have a synergistic effect in TGFβ1-initiated and ROS-dependent fibroblast-to-MF transition. So far, it was known, that TGFβ1 treatment increases the NAD(P)H oxidase activity in lung fibroblasts [13]. The non-phagocytic NAD(P)H oxidase produces primarily superoxide anions (O_2_^.−^) on the cytosolic side of the cell membrane [47], which may be subsequently dismutated to H_2_O_2_ [40,48].

In our study, the common flavoprotein inhibitor DPI and the selective NAD(P)H oxidase inhibitor apocynin [49] completely prevented both the TGFβ1-mediated expression of αSMA and the generation of ROS, excluding mitochondria or xanthine oxidase to be involved in the ROS-dependent transition of fibroblasts to MFs. Thus, it is likely to assume that the NAD(P)H oxidase is the only cellular source for the measured ROS in that context. The rate of NAD(P)H consumption by TGFβ1-treated cells resulted in two peaks indicating a biphasic activity of NAD(P)H oxidase. As demonstrated in non-phagocytic cells the late activation of NAD(P)H oxidase is characteristic for terminally differentiated MFs [13,40]. In contrast, we show that the rapid activation of the NAD(P)H oxidase is not part of the TGFβ1-mediated signalling and thus not essential for the fibroblast-to-MF transition. This is a novel aspect of TGFβ1-dependent NAD(P)H oxidase activation in skin fibroblasts. Furthermore, it was shown, that the generation of carbonylated proteins is regulated by NAD(P)H oxidase activity.

In addition, TGFβ1 stimulation of human lung fibroblasts resulted in a transient burst of ROS, which regulate the downstream events such as Ca^2+^ influx, MAPK activation and phosphorylation-dependent activation of AP-1 (activating protein-1), finally inducing interleukin-6 expression [50]. In our study, the MAPK inhibitor SB202190 completely abrogated the TGFβ1-dependent expression of αSMA. In human fibroblasts, it was shown, that treatment with TGFβ1 resulted in biphasic p38 activation [51]. Herein, the p38-peak at 12 h after TGFβ1 treatment is dependent on NAD(P)H oxidase activity. El-Remessy et al. also showed a ROS-dependent p38 activation induced by NAD(P)H oxidase [52].
